# Supplementary figures and images for: Depletion of WWP1 Increases Adrb3 Expression and Lipolysis in White Adipose Tissue of Obese Mice
Source: Int J Mol Sci. 2025 Apr 29;26(9):4219. doi: 10.3390/ijms26094219 (PMC12071688; doi:10.3390/ijms26094219)

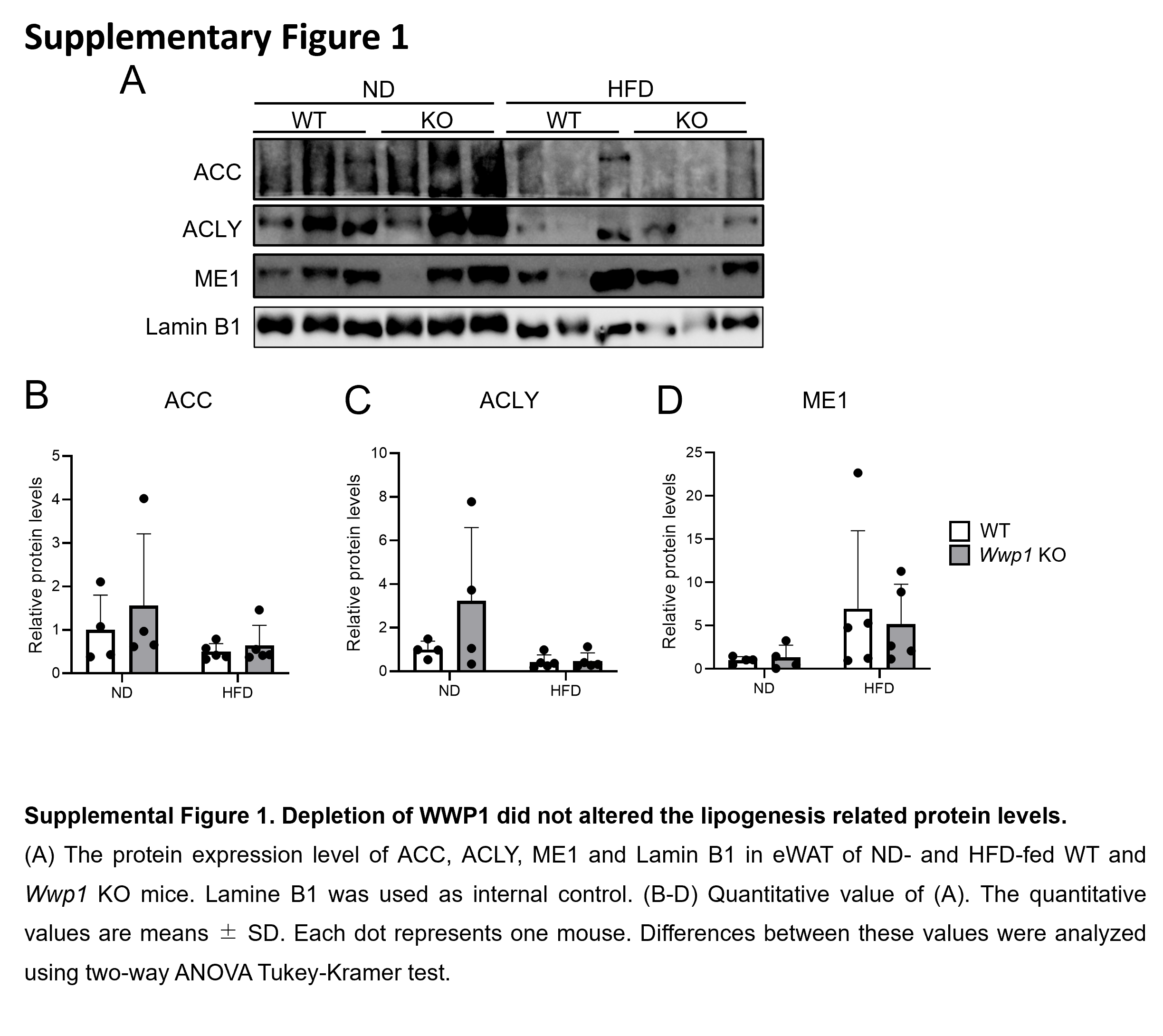

Supplement: Supplementary file 1 [file ijms-26-04219-s001.zip › Sup Fig 1_300dpi.tif]

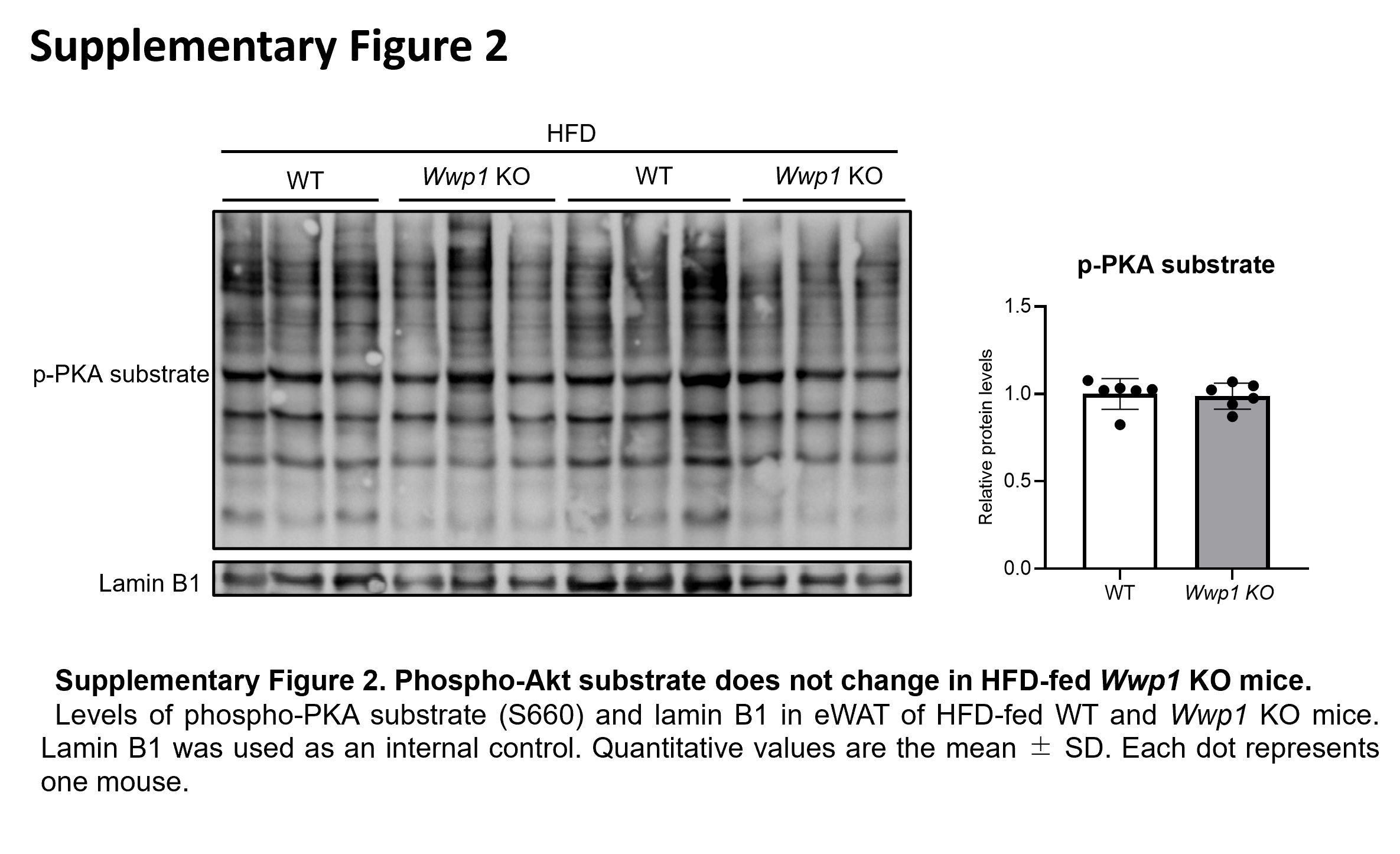

Supplement: Supplementary file 1 [file ijms-26-04219-s001.zip › Sup Fig 2_300dpi.tif]

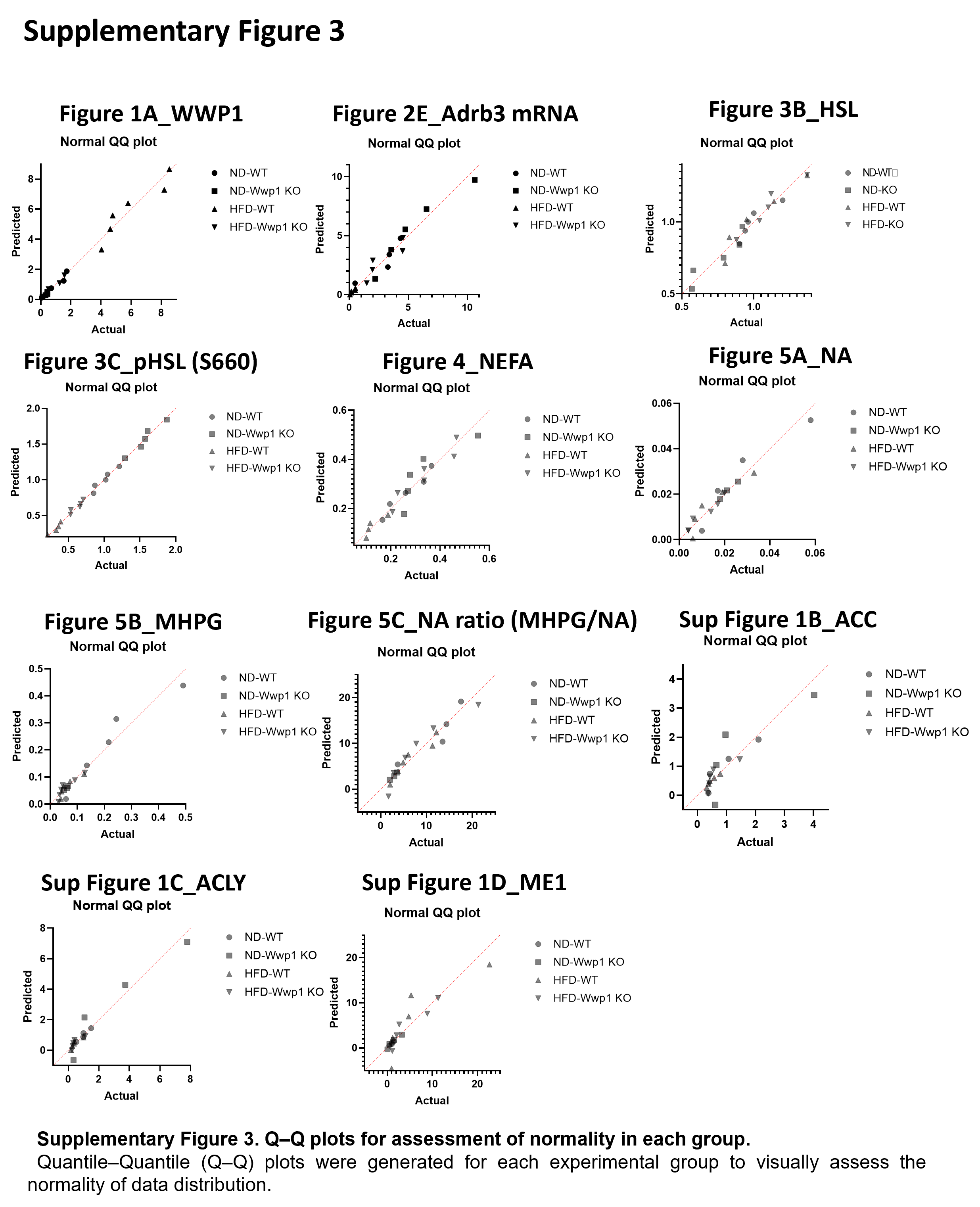

Supplement: Supplementary file 1 [file ijms-26-04219-s001.zip › Sup Fig 3_300dpi.tif]
